# Significance testing: Why does it prevail?

**DOI:** 10.1007/s10654-016-0220-0

**Published:** 2017-01-04

**Authors:** Anders Ahlbom

**Affiliations:** Department of Epidemiology, Institute of Environmental Medicine, Karolinska Institutet, Stockholm, Sweden

A report by Seliger et al. on statin use and risk of glioma prompted Greenland to write a letter-to-the-editor in which he again explains why lack of statistical significance must not be interpreted as lack of association [1, 2]. Greenland and colleagues have also stressed recently that a statistically significant association very well may be due to chance [3]. These two statements hold just the same regardless of whether the statistical significance judgment is based on if the *P* value is smaller than 5% or if the confidence interval excludes the no-effect value.

The consequences of dividing results into the two separate categories statistically significant and non-significant have been discussed extensively. The topic is part of the curriculum in courses and it appears in textbooks. Journals have had editorial comments and groups of experts with various tasks have provided guidance. Individual scientists have discussed this in commentaries like the current and in more comprehensive formats and there are other letters to the editor than Greenland’s that point to problematic use of the significance concept. For an extensive list of references, see a recent article in EJE [3].

Yet, the reporting style that points out whether associations are significant or not remains common. Although there is at most a thin marginal difference between a lower confidence bound of .99 and one of 1.01 we have all noticed disappointed faces when results start to appear and it becomes clear that figures don’t quite reach statistical significance and we have noted correspondingly happy faces in case of the opposite. A mechanism by which chance could be put out of the equation and the researcher freed to focus on systematic errors and biologic plausibility for assessment of causality would have been a great gift to the research community. But significance testing of null-hypotheses was not designed to serve this purpose. It was developed as a decision-making tool, and decisions are rarely made from the outcome of one single study.

A meta-analysis that offers new insights into ways of reporting study results is published in the current issue of European Journal of Epidemiology [4]. It is an attempt to estimate trends in usage of *P* values, significance tests, and confidence intervals in close to 90,000 articles published in five general medical journals and in seven epidemiology journals. The basis is the computerized abstracts in PubMed, which allows for the large study size but limits the information to the wordings in the abstract. The key findings are that confidence intervals presented in their own right and not as proxies for statistical tests are becoming more common, particularly in epidemiology journals. Although significance testing is becoming less popular in most epidemiology journals and some widely read medical journals, it is still very common in some prominent medical journals. While these results signal an improvement over time and a rather positive trend, particularly among epidemiology journals, it is worth noting that still only about 40% of the articles in epidemiology journals rely solely on confidence intervals for assessing precision in the reported estimates, based on what shows up in the abstracts. In the selected medical journals this figure was about 20%.

A few things are immediately clear. First, editorial policy plays a role as evidenced by the position that Epidemiology takes. Confidence intervals have always been the predominant mode of reporting in this journal as the result of an editorial policy that was in place from the start of the journal. Second, there is a clear difference between epidemiology journals and medical journals with considerably more reliance on confidence intervals in the epidemiology journals; the data don’t allow a comparison restricted to reports of epidemiological studies. Third, the prevalence of statistical significance testing varies across medical journals and in particular prevails over time in the high impact journals JAMA, NEJM, and Lancet.

Thus, this meta-analysis informs about the prevalence and trends in usage of significance testing, but we still don’t know why this so often is the reporting style of choice. We also don’t know the reason for the differences in this respect between types of journal and between individual journals. A list of candidates for explanation is provided below. These explanations are based on a blend of different factors including compliance with perceived or real expectations from the surrounding research community, on convenience on the side of the researcher when reporting results, and on ignorance.

This is the list:

1. If the editor of the intended journal requests statements as to whether or not results are statistically significant these will most likely be provided.
2. Indeed, it may even suffice that the editor is anticipated to request such statements for them to be provided.
3. Most researchers do not want to be seen as exaggerating their own findings and that makes some researchers particularly careful with associations that are non-significant and they may therefore be anxious to declare whether results pass the cut off or not.
4. A researcher may think that after all, a significant result is stronger than one that isn’t and a non-significant result weaker than one that is. Also when confidence intervals are used as the mood of reporting the researcher may therefore choose to emphasize if the confidence interval includes the null or not (although obvious to the reader anyway) by referring to the results as significant or non-significant.
5. Describing the results of a study becomes easy with statistical significance language because it provides standardized phrases. Without this language the researcher must describe the findings with own words.
6. Research typically results in more than one result, and certain choices need to be made by the researcher when the results are reported and discussed. Choosing the ones to report or highlight is made easy if based on statistical significance.
7. A somewhat similar situation may arise with meta-analysis. The researcher who conducts the meta-analysis may find it convenient to classify the selected studies into whether their results are significant or not, regardless of how they were presented in the original article. The meta-analysis may then be based on a count of the positives and the negatives. This may well result in that a set of positive studies, but with confidence intervals including the null, are considered as all negative, although taken together they could be overwhelmingly positive.
8. It is conceivable that some statistical courses cover various approaches to random variation in a neutral tone and commenting that different methods to a certain extent are interchangeable, but without a proper discussion of relevance and interpretation. If such courses are not accompanied by method-oriented epidemiology training the researcher is left alone and in the lack of guidance may decide to go for significance testing.
9. A researcher without formal training in epidemiology, perhaps in a clinical setting, who conducts an epidemiological study, may turn to a biostatistician for assistance when analyzing the data. The biostatistician may be indifferent as to the reporting style and prepared to deliver whatever is requested. The two may together decide to go for significance tests.
